# Pancreatic Ductal Adenocarcinoma: Update of CT-Based Radiomics Applications in the Pre-Surgical Prediction of the Risk of Post-Operative Fistula, Resectability Status and Prognosis

**DOI:** 10.3390/jcm12237380

**Published:** 2023-11-28

**Authors:** Giulia Pacella, Maria Chiara Brunese, Eleonora D’Imperio, Marco Rotondo, Andrea Scacchi, Mattia Carbone, Germano Guerra

**Affiliations:** 1 Department of Medicine and Health Science “V. Tiberio”, University of Molise, 86100 Campobasso, Italy; giulia_pacella@yahoo.it (G.P.);; 2 Radiology Unit, “A. Cardarelli” Hospital, 80131 Campobasso, Italy; e.dimperio@studenti.unimol.it; 3 General Surgery Unit, University of Milano-Bicocca, 20126 Milan, Italy; 4 San Giovanni di Dio e Ruggi d’Aragona Hospital, 84131 Salerno, Italy; mattia.carbone@sangiovannieruggi.it

**Keywords:** pancreatic ductal adenocarcinoma, radiomic, artificial intelligence, presurgical evaluation, POPF, prognosis

## Abstract

Background: Pancreatic ductal adenocarcinoma (PDAC) is the seventh leading cause of cancer-related deaths worldwide. Surgical resection is the main driver to improving survival in resectable tumors, while neoadjuvant treatment based on chemotherapy (and radiotherapy) is the best option-treatment for a non-primally resectable disease. CT-based imaging has a central role in detecting, staging, and managing PDAC. As several authors have proposed radiomics for risk stratification in patients undergoing surgery for PADC, in this narrative review, we have explored the actual fields of interest of radiomics tools in PDAC built on pre-surgical imaging and clinical variables, to obtain more objective and reliable predictors. Methods: The PubMed database was searched for papers published in the English language no earlier than January 2018. Results: We found 301 studies, and 11 satisfied our research criteria. Of those included, four were on resectability status prediction, three on preoperative pancreatic fistula (POPF) prediction, and four on survival prediction. Most of the studies were retrospective. Conclusions: It is possible to conclude that many performing models have been developed to get predictive information in pre-surgical evaluation. However, all the studies were retrospective, lacking further external validation in prospective and multicentric cohorts. Furthermore, the radiomics models and the expression of results should be standardized and automatized to be applicable in clinical practice.

## 1. Introduction

Pancreatic ductal adenocarcinoma (PDAC) is one of the leading causes of cancer-related deaths worldwide. By 2030, it is predicted to become the second leading cause of mortality for cancer [1,2,3].

As reported in the surgical management of other gastrointestinal cancers, such as metastatic colorectal cancer [4,5,6,7,8], due to the late presentation of clinical symptoms, only 15–20% of patients are candidates for curative intent pancreatectomy at diagnosis [9,10,11,12].

However, in pancreatic cancer, resectable upfront the 5-year survival is low, estimated at 25–30% [13,14,15,16]. Moreover, undetectable micro-metastases can develop once the primary tumor manifests in imaging, giving the appearance of resectable disease with standard diagnostic protocols even when there is indeed systemic spread [17,18,19,20,21].

In most clinical practice recommendations and guidelines, contrast-enhanced-CT (CECT) is the first-line tool for the detection, staging, and therapeutic determining of PDAC, representing the gold standard imaging method in patients undergoing surgery [22,23,24,25,26,27,28].

CT can be also associated in selected patients with MRI, to evaluate the tumor invasion or contact with bordering vascular structures such as vein porta, mesenteric vein, and mesenteric artery. However, the diagnostic sensitivity of CT and MRI of vascular invasion is approximately 71–74% and specificity is 97% [29,30].

The typical appearance of PDAC at CT imaging is a hypoenhancing infiltrative mass, hypodense in the pancreatic phase (late arterial phase) with delayed enhancement in later phase images because of central necrosis and desmoplastic reaction [31]. Distal pancreatic duct dilatation and atrophy are related to an obstruction in the head of the pancreas [32]. Pancreatic lesions may also be secondary to renal cancer and other gastrointestinal cancers, but they are rare and do not undergo surgical treatment [33].

In many fields of radiology, to improve the diagnostic performance of the standard radiological protocols, several studies have already proposed radiomics tools. Radiomics refers to a non-invasive methodology able to extract and analyze data from radiological images. In this way, radiomics process images through numerical features that may be related to a clinical pattern undetectable to the naked eye [34,35].

Those features usually include signal intensity, shape, texture, and higher-order texture features. One of the most important clinical-relevant applications of radiomics is the ability to help predict the survival of cancer patients in a preoperative setting [36,37,38].

Many artificial intelligence methodologies have been proposed using CT or MRI imaging, with the best results in gastrointestinal tumors, gangliomas, lung cancer, and head and neck cancer [39,40,41,42,43,44,45,46]. Concerning PDAC, radiomics have been applied to the diagnosis, to the pre-operative evaluation, to response to therapy, and to predict prognosis and complications after treatment [47,48,49,50,51].

Therefore, several authors have proposed radiomics models for early preoperative risk stratification, including studies addressed to main topics: preoperative prediction of resectability status, preoperative prediction of postoperative pancreatic fistula (POPF), and preoperative prediction of overall survival after treatment [52,53,54,55].

Radiomics approach to medical imaging usually consists of automatic or manual segmentation of the ROI, followed by feature extraction, feature selection and reduction, statistical analysis, and model creation [56,57,58,59,60].

Then, machine learning methods are used to associate the features with the given clinical task. Deep learning methods consist of automated learning of relevant features, avoiding the requirement of human interventions [61,62,63,64,65].

Going further, 3D convolutional neural network (CNN)-based prognosis models have shown good performances in outcome prediction, actually of lung cancer and gliomas [66,67,68,69,70,71].

The aim of this review is to report on the results of several studies and the real application of radiomics in clinical practice in the large field of PDAC diagnosis, therapy, and prognosis. The included studies address the following main topics: preoperative prediction of resectability status and preoperative prediction of POPF.

### 1.1. Resectability Status

According to the 2021 NCCN guidelines, the resectability status of pancreatic cancer is differentiated by contrast-enhanced CT scanning into four classes: resectable, borderline resectable, locally advanced/unresectable, and metastatic pancreatic adenocarcinoma. Different resectability statuses reflect different management and prognosis [72,73,74,75,76].

According to the guidelines, a resectable PDAC is characterized by no evidence of distant metastases, no CT signs of superior mesenteric vein or portal vein distortion, and clear fat planes around celiac axis, hepatic artery, and superior mesenteric artery [77]. Since surgical R0-resection may be a potentially curative treatment option for patients with PDAC, accurate quantitative preoperative prediction of primary resectable tumors is required [78,79,80,81,82,83].

Borderline resectable PDAC definition includes vascular invasion of the superior mesenteric vein and portal vein, gastroduodenal encasement up to the hepatic artery without extension to the celiac axis, and tumor contact <180° of the circumference of the vessel wall of the superior mesenteric artery.

The correct timing of surgery is still under debate in spite of neoadjuvant therapy in PDAC with resectable or reconstructable vessels involved. A study conducted by Kelly et al. showed no significant difference in survival among patients undergoing R0 resection with vascular reconstruction comparable to that observed in upfront resectable PDAC [84].

Locally advanced pancreatic cancer (LAPC) is defined as a non-metastatic cancer, with an extensive vascular involvement that does not allow a radical resection [85]. At the time of diagnosis, one-third of patients present with locally advanced pancreatic cancer (LAPC) [86,87,88,89,90].

The actual standard of care for LAPC consists of multiagent chemotherapy regimens neoadjuvant treatment, with improved survival compared to mono-chemotherapy schedules, and then evaluating the response to therapy [86,91,92,93,94,95,96].

In selected patients, there is the possibility of maximizing the benefits of the oncological treatment integrating radiation therapy (RT) approaches both in locally advanced patients and after R0 resection as adjuvant therapy [97,98,99,100,101].

When the response to neoadjuvant therapy consists of an important regression of disease, the surgical treatment can improve overall survival in LAPC [102,103,104,105,106].

However, after neoadjuvant therapy, the restaging CT imaging has a low diagnostic performance to evaluate the residual tumor-burden, due to the difficulty in distinguishing neoplastic tissue from fibrous scar or inflammation [61,107,108,109,110]. Radiomics could play a decisive role in integrating traditional morphological parameters in predicting surgical resection, both in primarily resectable patients and even in locally advanced pancreatic cancer (LAPC) after neoadjuvant treatment [111].

### 1.2. Post-Operative Pancreatic Fistula

Postoperative pancreatic fistula (POPF) is a severe complication caused by pancreato-enteric anastomotic leakage, that occurs in about 10–30% of patients undergoing pancreatic head resection for surgical treatment of pancreatic cancer [112,113,114,115,116,117,118].

According to the 2016 POPF definition of the International Study Group on Pancreatic Fistula (ISGPF), an asymptomatically elevated level of drain amylase is only a mild form of biochemical leak, while the clinically relevant (CR-) POPF is the most challenging complication after pancreatoduodenectomy (PD) in terms of morbidity/mortality rates [119,120,121].

**Figure 1:**
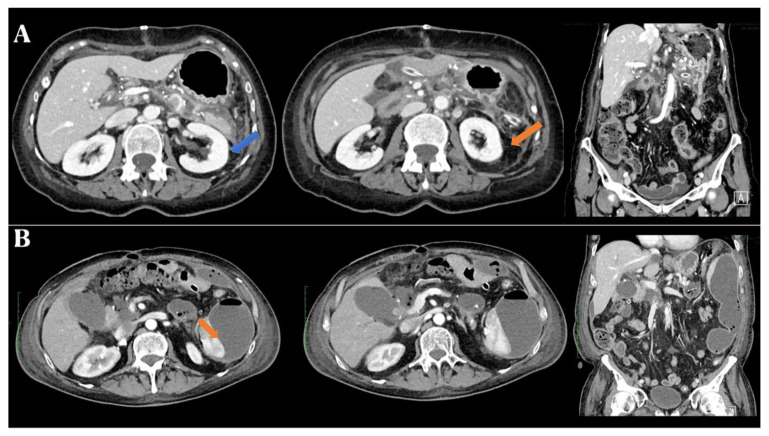
Computed Tomography images of patients who underwent pancreatic surgery. (**A**) Post-Operative Pancreatic Fistula Grade A according to International Study Group of Pancreatic Fistula (ISGPF); (**B**) Post-Operative Pancreatic Fistula Grade B according to International Study Group of Pancreatic Fistula (ISGPF). The blue arrow indicates the distal pancreas; the orange arrow indicates the peripancreatic fluid in (**A**) and the organized fluid collection in (**B**).

In the literature, different grades of POPF are described. Grade A has no clinical impact, as it does not imply a longer hospital stay or interventional procedures (Figure 1).

Grade B is characterized by an increase in amylase activity from the drain and it has a clinical impact on the patient (Figure 1). Interventional management is required for drain collection through US or CT-guided or endoscopic procedures. In case of POPF-related hemorrhage, pseudo-aneurism transfusions and/or angiography are required.

Grade C fistula is considered an evolution of grade B when reoperation is needed due to organ failure or clinical instability although an interventional procedure has taken place. Reoperation is related to high morbidity and mortality [122,123].

The current gold standard of assessing CR-POPF risk is based on intraoperative factors: pancreatic texture, pancreatic duct width, and BMI are established risk factors [124,125,126,127,128].

A validated and used score is the Fistula Risk Score (FRS), a 10-point scale that includes four parameters: small-sized pancreatic duct, palpatory soft glandular texture, underlying high-risk pathology (chronic pancreatitis or pancreatic adenocarcinoma) and intraoperative blood loss [129,130,131,132].

A more accurate risk assessment of developing POPF can impact the pre-surgical decision-making process and lead to better perioperative management of patients [114,133,134,135,136].

To our knowledge, there is no risk score based on diagnostic imaging, as it happens for acute pancreatitis. As AI-based models are strongly predictive, radiomic features from preoperative CT imaging related to clinical factors can help identify patients at high risk for postoperative pancreatic fistula following pancreatic head resection [137,138].

### 1.3. Prognosis

The common clinical-pathological factors determining prognosis and overall survival of patients who undergo surgical resection of PDAC include tumor size, lymph node involvement, margin status of surgically resected tumor, carbohydrate antigen 19-9 (CA19-9) level, and the adjuvant chemotherapy received [15,139,140,141,142,143].

Several previous studies have explored how preoperative CT texture radiological features correlate with overall survival [144,145,146,147,148,149,150].

## 2. Methods

We searched the PubMed database (US National Library of Medicine, http://www.ncbi.nlm.nih.gov/PubMed) using the subsequent keywords: [pancreas AND (cancer OR adenocarcinoma OR tumor OR neoplasia OR malignancy) AND (artificial intelligence OR radiomics OR deep learning OR machine learning OR neural network) AND (prognosis OR treatment OR surgery OR complications OR fistula OR outcome OR survival)].

Papers had to have been published no earlier than January 2018. Articles were first chosen based on title and abstract, but a review of the available full text was necessary in order to include the article. Clinical studies (retrospective analysis, case series, prospective cohort study) were reviewed. Case reports, reviews, comments, or letters to editors were excluded.

## 3. Results

We recognized 301 pertinent papers based on a review of titles and abstracts, then we narrowed them down to 11 full-text articles concerning specific radiomic tools based on CT preoperative imaging for detection, treatment strategy, and survival prediction of PDAC. Articles first excluded for title and abstract were reviews or case reports, or they did not address PDAC or CT as the chosen imaging method.

Of the 11 most pertinent original articles included, 4 were about resectability status prediction, 3 were about preoperative POPF prediction, and 4 were about survival prediction [61,137,151,152,153,154,155,156,157,158,159].

## 4. Discussion

### 4.1. Resectability Status

Although there is careful selection of resectable PDAC patients, the recurrence rate after upfront surgery can be very high, especially within 12 months from the index procedure, resulting in poor prognosis [160,161,162]. Upfront surgery cannot be the best treatment approach for patients who are not primary resectable, who could instead benefit from neoadjuvant chemotherapy [163,164].

The challenge of the retrospective study of Palumbo et al. [153] was to combine clinical, radiological, and radiomic features to build and validate a machine-learning-based model of preoperative risk stratification of disease recurrence in 12 months in distant sites after upfront pancreaticoduodenectomy. In a retrospective study of 147 patients who underwent upfront surgery, 182 radiomics features were extracted from preoperative clinical and radiological CT-based data, and the most discriminant features were selected. Clinical (serum level of CA19-9), radiological (necrosis), and radiomic (SurfAreaToVolumeRatio) features were significantly associated with early recurrence. The model combining these three variables performed well in the training cohort (*p* = 0.0015, HR = 3.58, 95%CI = 1.98–6.71) and was then confirmed in the validation cohort (*p* = 0.0178, HR = 5.06, 95%CI = 1.75–14.58). There was a significant statistical difference in survival curves between low and high-risk patients (*p*-value < 0.0001). As a result, this model may help to better define resectability status and to identify patients who would benefit from neoadjuvant chemotherapy instead of upfront surgery. Patients considered at low risk of early recurrence may represent the group potentially suitable for primary resection.

In the case of patients with locally advanced PC (LAPC) at the time of diagnosis, resectability is usually reassessed after neoadjuvant treatment by CT-scan imaging, but after radiotherapy CT cannot discriminate between post-therapy fibrosis, locoregional edema, inflammatory changes, and visible tumors [165,166].

The aim of the study of Rossi et al. [152] was to assess the capability of CT-based radiomic features to predict the response to therapy and the resectability in LAPC treated with both neoadjuvant chemotherapy and radiotherapy. In a retrospective study of 71 patients who underwent inductive chemotherapy followed by ablative radiotherapy, a predictive model of resectability status after treatment was developed and validated. ROI was defined on the gross tumor volume and 1655 radiomic features extracted from CT at primary staging integrated with clinical data. The median Area Under the Curve (AUC) was 0.862 (95% CI: 0.792–0.921) for the training set and 0.853 (95% CI: 0.706–0.960) for the validation set. The validated model was applied to the entire dataset and 4 futures were selected to build the model with predictive performance as measured using an AUC of 0.944 (95% CI: 0.892–0.996). In model-building, clinical data was not significant enough to separate resected patients from not-resected patients, therefore the radiomic model alone achieved the same results. This means that radiomics represents a fundamental tool that can be used to decode information that cannot be obtained from the naked-eye view of CT images or other clinical data alone [167].

Furthermore, in patients with unresectable advanced disease undergoing chemotherapy, Salinas et al. [156] suggest that, given the biological overlap between patients appearing resectable and patients appearing non-resectable on CT, it is possible that prognostic features in resectable PDAC may also be prognostic in unresectable PDAC. From a cohort of 108 patients enrolled in a prospective chemotherapy trial, the pre-treatment CT scans were used as a test cohort for 2 previously published prognostic radiomic features in resectable PDAC (Sum Entropy and Cluster Tendency with square-root filter (Sqrt)). Then the performance of these 2 radiomic features for the prediction of overall survival (OS) and time to progression (TTP) was assessed using Cox proportional hazard models. Sqrt Cluster Tendency was significantly associated with outcome both in terms of overall survival (HR of 1.27 for primary pancreatic tumor plus local nodes; CI: 1.01–1.6, *p*-value 1⁄4 0.039) and of Time to progression (HR of 1.25; CI:1.00–1.55, *p*-value 1/4 0.047). As a result, the CT radiomic feature Sqrt Cluster Tendency, previously demonstrated to be prognostic in resectable PDAC, remained a significant prognostic factor for OS and TTP in a test set of unresectable PDAC patients, despite the cohort being used as a test set for 2 previously validated radiomics parameters, and not as a training set. This suggests that maybe there are similarities in radiomic features of both early and advanced disease reflective of aggressive biology [156].

In the pre-operative evaluation of resectability, an accurate study of tumor biology markers is required to correctly stage the disease [168,169,170,171,172].

Finally, the work by Yao et al. [61] suggests a new multi-task CNN framework for cancer survival prediction by simultaneously predicting the tumor resection margins for PDAC patients.

Both tumor attenuation and resection margin are associated with patient’s clinical outcomes: hypo-attenuating mass indicates a low stromal fraction, related to a worse clinical outcome; while R0 resection is usually associated with relatively long-term survival, and R1 resection may have a high recurrence chance [173].

The Yao et al. [61] radiomics model, called CE-ConvLSTM, can derive the tumor attenuation signatures or patterns from patient CE-CT imaging studies.

Dynamic Contrast-Enhanced Computed tomography (DCE-CT) remains the primary initial imaging modality of choice for pancreatic cancer diagnosis. It plays a major role in depicting, staging, patient management, and evaluating PDAC resectability [174,175].

In fact, the second aim of the study was the evaluation of resection margin status in the form of tumor–vascular relationships, which is known to be strongly related to the overall survival of PDAC patients [176,177,178,179,180].

The model used a self-learning approach for automated pancreatic and peripancreatic anatomy segmentation, without requiring any annotations on PDAC datasets, and then was employed into a multi-task convolutional neural network (CNN) to accomplish both tasks of survival outcome and margin prediction.

The use of both CE-ConvLSTM and self-learning segmentation to encode dynamic tumor attenuation patterns and pancreatic anatomies significantly outperforms in terms of overall survival prediction when compared with AJCC 6th edition (C-index of 0.667 vs. 0.613; AUCs of 0.718–0.707 vs. 0.665–0.622 of pT stage). Moreover, this marker is obtained pre-operatively while the AJCC staging for patients who underwent resection is post-operative.

Both survival and resection margin predictions can serve as strong prognostic and predictive quantitative biomarkers for subgroup patients after staging by the well-established pathological TNM or tumor size criteria.

The marker proposed by Yao et al. [61] promises to guide PDAC treatment selection before surgery while likely having a higher prediction accuracy than the post-operative staging system. Possible improvements could be a more sophisticated representation of the tumor-vessel relationship, CT-based lymph node metastasis detection, and integration with other established clinical and pathological variables such as CA19-9 and AJCC staging system.

To summarize, in the field of resectability status prediction, radiomics tools have obtained promising results both in the pre-operative prediction of recurrence after upfront surgery in resectable tumors and in the prediction of resectability in LAPC after chemo-radiotherapy. Furthermore, the model proposed by Yao et al. is very promising to guide the surgical treatment recommendations of patients with primary resectable PDAC tumors.

### 4.2. Post-Operative Pancreatic Fistula

For the prediction and early detection of POPF, radiomic features extracted from preoperative CT imaging, in combination with clinical factors, can help to identify patients at high risk for postoperative pancreatic fistula following pancreatic head resection [137,181,182,183].

Bhasker et al.’s purpose [137] was a CR-POPF preoperative prediction model based on preoperative CT-based radiomic features, including volumes of annotated intra- and peripancreatic structures integrated with preoperative clinical data and compared with established CR-POPF clinical risk measurements.

The predictions of this pre-operative signature showed a strong correlation with the intraoperative updated alternative fistula risk score (ua-FRS), which is the clinical gold standard for intraoperative CR-POPF risk stratification. The combined radiomic and clinical signature built on 108 patients having undergone pancreatoduodenectomy achieved an AUC of 0.86 and a balanced accuracy score of 0.76 on validation data. Another study by Capretti et al. proposed a machine-learning model built on 100 patients, 20 of them with POPF. The results achieved were a specificity of 0.824 (0.133) and a sensitivity of 0.571 (0.337), with an AUC of 0.807 (0.155) [158].

These results indicate that the proposed combined radiomic and clinical preoperative evaluation can be performed for CR-POPF risk stratification.

Furthermore, several AI approaches to the quantitative preoperative CT assessment of POPF have been evaluated, with the most promising results being from Machine Learning (ML) models and Deep-Learning (DL) models.

ML could discover intricate structures in large datasets by using a backpropagation algorithm, making it useful to identify latent variables and to develop a risk prediction platform to optimize individual treatment decisions [184]. In a recent retrospective study by Han et al. [185], from 1769 patients who underwent surgery, 38 variables were inserted into AI-driven algorithms to make the risk prediction platform, with a maximal AUC using NN of 0.74. AI algorithm identified 16 risk factors for POPF: pancreatic duct diameter, body mass index, preoperative serum albumin, lipase level, amount of intraoperative fluid infusion, age, platelet count, extrapancreatic location of tumor, combined venous resection, co-existing pancreatitis, neoadjuvant radiotherapy, ASA score, sex, soft texture of the pancreas, underlying heart disease, and preoperative endoscopic biliary decompression [129,185,186,187].

On the other hand, the automated DL could reflect the morphologic features of the pancreatic duct, remnant pancreatic tissue volume, and parenchymal fibrosis. The Deep Learning Score (DLS) is particularly helpful for patients with intermediate FRS risks in predicting the risk of CR-POPF. A DLS has been described as a highly accessible DL model of routine patient examination, that automatically generates features from preoperative CT scans with excellent performance compared to CT classifiers cited in the literature (remnant pancreatic volume, MPD diameter, pancreatic width or thickness) [157,188,189,190,191,192,193].

In the study by Mu et al. [157], a group of 513 patients who underwent surgery was retrospectively collected and formed a training (70%) and a validation dataset (30%) randomly. A CNN was trained and generated a DLS to identify the patients with a higher risk of CR-POPF preoperatively using CE-CT images. As a result, compared to FRS, the DLS offered significantly greater predictability both in training (AUC:0.85 [95% CI, 0.80–0.90]), and in validation groups (0.81 [95% CI, 0.72–0.89]) with challenging results for intermediate-risk patients (FRS: 3–6), where DLS showed significantly high accuracy.

To summarize, in the field of preoperative prediction of POPF, the actual results indicate that the preoperative combination of CT-radiomic features and clinical preoperative evaluation can perform CR-POPF risk stratification better than only clinical assessment. An ML-based AI algorithm has been developed, with 16 risk factors for POPF identified, demonstrating its added value in the identification of latent variables. The study of DL-based methods suggests that DLS could be particularly helpful for patients with intermediate FRS risks in predicting the risk of CR-POPF.

Minimally invasive surgery is now common in clinical practice not only in general surgery but also in oncological pathologies [28,194,195,196,197,198,199,200,201,202,203].

Referral and peripheral centers have been developed and have participated in many hub-and-spoke learning programs to ensure the standard of care [204,205,206,207].

In this era, several studies have reported the usefulness and feasibility of pancreatic resection through laparoscopy or a robotic approach.

However, the validation and estimation of complications with different methods is still under review by randomized control trial.

There is also the conflict concerning the surgical technique used in peripheral centers of proceeding to the distal pancreatic duct occlusion in spite of the anastomosis [208]. To our knowledge, there are no studies proposing radiomic models to choose among different surgical approaches.

Radiomics can be used to help surgeons and clinicians choose the most appropriate approach for each patient in order to personalize not only the timing of resection but also the approach, predicting the different risks of complications.

### 4.3. Prognosis

Nomogram-based overall survival prediction is based on tumor differentiation, margins, T stage, and number of positive nodes (C-indexes up to 0.64) [209,210,211].

Presently, the pre-operative prognostic factors of PDAC are related to the CT and MRI evidence of nodal metastases and secondarism and T-stage, and also to the vascular invasion of the hepatic and mesenteric arteries and mesenteric and portal veins [212]. After surgery, the prognostic factors are also related to margin evaluation, perineural and lymphovascular invasion, and tumor grading [213].

Park et al. [154] evaluated the prognostic utility of preoperative CT radiomics features in predicting the postoperative survival of patients with PDAC, by the selection of optimal radiomics features including tumor heterogeneity and texture of the whole pancreas to capture peritumoral phenotype [214,215,216]. In a retrospective study of 153 patients who underwent pre-operative CT, a total of 478 radiomics features were extracted from tumors and 11 extra features were computed from pancreas boundaries. The 10 most relevant features were selected to distinguish the high- and low-risk groups according to survival time. High-risk patients were those who survived less than 1 year and low-risk patients were those who survived more than 3 years from the date of initial diagnosis. Survival analysis was performed considering clinical parameters both with and without the addition of the selected features. The 10 most relevant radiomics features showed 82.2% accuracy in the classification of high-risk versus low-risk groups. The C-index of survival prediction with clinical parameters alone was 0.6785.

The addition of CT radiomics features Improved the C-index to 0.7414. The high-risk group showed a higher classification performance, with 86.4% accuracy, compared with the low-risk group, with 78.3% accuracy. In conclusion, the addition of CT radiomics features to standard clinical factors improves survival prediction in patients with PDAC [145,154,217,218,219,220].

Other recent studies about survival prediction in PDAC have reported the better performance of a combination of radiomics CT features and basic clinical features [144,221,222,223,224,225,226].

Among the evaluation of significant clinical features, the evaluation of frailty surely impacts hospitalization and prognosis [227,228,229,230,231,232,233,234,235,236].

In particular, body composition features measured by CT imaging are gaining increasing attention in clinical oncology, due to an identified prognostic adverse potential in patients with breast cancer, renal cell carcinoma, and lung and esophageal cancer [234,237,238,239,240,241,242]. In this framework, the aim of the study by Shi et al. [155] was to develop a radiomics model based on radiomic signature, clinic-pathologic factors, and body composition measures to estimate the overall survival (OS) in patients with PDAC. This study also aimed to evaluate the model’s incremental value to the traditional AJCC TNM staging system, which is unable to consider that tumors have differing genetic, cellular, and behavioral characteristics even in same-stage patients [155].

For radiomics signature construction, the most significant features were extracted from CE-CT. Then, five independent variables were selected for the radiomics model: radiomics signature, CA19-9, skeletal muscle index, histologic grade, and postoperative chemotherapy. The predictive performance of the radiomics-based model was superior to the one achieved by the clinical model alone and to the AJCC TNM staging system (C-index 1/4 0.73; all *p* < 0.05). Patients were stratified into high-risk and low-risk groups by the radiomics model. Moreover, in their study, the multivariate Cox analysis identified body measurements as independent risk factors. This finding is in keeping with the results of other studies in which sarcopenia is an adverse prognostic indicator for PDAC [243,244,245,246,247].

On another hand, the grade of portal venous vascular involvement in patients with PDAC may be a prognostic indicator of survival, but is only a bidimensional measurement of the relationship between the tumor and the portal vein [248,249].

Several studies about prognosis prediction based on conventional radiological or radiomic features such as intensity, texture, and morphology, have not used reproducible methods, which may reduce clinical applicability because radiomic intrinsic evaluation of soft tissue tumors has low reproducibility of intensity and texture features and also among different segmentation methods [221,250,251,252,253,254,255,256,257].

The integration of radiomic features and vascular involvement could represent a more reproducible and applicable strategy in survival prediction [258,259].

The aim of the study of Rigiroli et al. [151] was to develop and evaluate task-based radiomic features extracted from the mesenteric-portal axis for the prediction of survival and response to neoadjuvant therapy in patients with PDAC. In a retrospective study of 107 patients who underwent neoadjuvant therapy followed by surgery for PDAC, task-based shape radiomic features were extracted and analyzed from the mesenteric-portal axis before (CTtp0) and after (CTtp1) neoadjuvant therapy. These features aimed to assess MPA shape, MPA narrowing, changes in shape and diameter between CTtp0 and CTtp1, and length of the MPA segment affected by the tumor.

A Cox proportional hazards model that included three selected radiomic features (Eccentricity mean tp0, Area minimum value tp1, and Ratio 2 minor tp1) plus clinical information showed an integrated AUC of 0.72 for prediction of survival, and a better fit compared to the model with only clinical information.

Finally, according to Kim et al. [260], ML models, compared to Cox models for survival prediction in resected PDAC, have not shown a significant improvement.

Two ML methods (RSF and SVM) were compared to Cox models, obtained from two nationwide databases (SEER and KOTUS-BP), and then also from mixed datasets.

Compared with the Cox model, the performance of the ML survival models was not significantly improved. This is probably due to the poor number of shared variables between the datasets, which might have been too few to maximize the usefulness of ML methods. ML methods are useful to analyze more complex and nonlinear associations among high-dimensional variables such as genetic information [260]. ML survival models could probably recommend whether a patient should receive treatment or not [261,262].

To summarize, in the field of survival prediction, preoperative CT scans show the global status of pancreatic parenchyma and tumor texture, and radiomics features extracted from tumors and from the non-neoplastic pancreas can be used to improve the survival prediction models of patients who undergo surgery for PDAC. However, more information could also be considered for further studies, for example, the correlation with detailed types of adjuvant therapy, and postoperative clinical parameters, for a better prediction of overall survival [144].

Additionally, a prognostic model based on radiomics signature, clinic-pathologic features, and also on body composition measures performed better than the TNM staging system and may facilitate optimal personalized management after resection [263].

Despite this limitation, the established radiomics model has the potential to be used as a biomarker for risk stratification of survival outcomes in patients with PDAC and thus may serve as a tool to guide individual postoperative care. Moreover, radiomic features extracted from the mesenteric-portal axis play a challenging role in providing additional preoperative information for the prediction of survival, even though the involvement of arteries is crucial to survival [264].

## 5. Conclusions

AI models of image analysis for risk prediction of several cancers have shown huge performance, surpassing the accuracy and time efficiency of traditional procedures [265].

Radiomic information, so, may complement clinical and morphological parameters in many fields, but advances in artificial intelligence must be interpreted with caution. Unfortunately, virtually all studies about AI were made retrospectively, and more research is needed to make sure than the use of AI provides equivalent results in real-world prospective studies [266].

In all the fields of applications of radiomics concerning treatment, in particular pre-operative evaluation and resectability, surgical complications, and prognosis prediction, we have not found any prospective study or prospective validation of the model. Therefore, the main limitation of the reported models is the need for testing in clinical practice.

Moreover, the application to clinical practice is still very limited, due to methodological issues such as different imaging parameters delineation, intra and interscanner variability, and need for clinical interpretation of any radiomic signature. Radiomics models and their expression of results should be standardized and automatized to be applicable in clinical practice [267,268].

## Data Availability

The data supporting the reported results can be found in the references.
